# Nanoparticle-Mediated Seed Priming Improves Germination, Growth, Yield, and Quality of Watermelons (*Citrullus lanatus*) at multi-locations in Texas

**DOI:** 10.1038/s41598-020-61696-7

**Published:** 2020-03-19

**Authors:** Pratibha Acharya, Guddadadarangavvanahally K. Jayaprakasha, Kevin M. Crosby, John L. Jifon, Bhimanagouda S. Patil

**Affiliations:** 1 Vegetable and Fruit Improvement Center, Department of Horticultural Sciences, Texas A&M University, 1500 Research Parkway, College Station, TX 77845 United States; 2 Department of Horticultural Sciences, Texas A&M AgriLife Research and Extension Center, 2415 E Hwy 83, Weslaco, TX 78596 United States

**Keywords:** Physiology, Plant sciences

## Abstract

Seed priming uses treatments to improve seed germination and thus potentially increase growth and yield. Low-cost, environmentally friendly, effective seed treatment remain to be optimized and tested for high-value specialty crop like watermelon (*Citrullus lanatus*) in multi-locations. This remains a particularly acute problem for triploids, which produce desirable seedless watermelons, but show low germination rates. In the present study, turmeric oil nanoemulsions (TNE) and silver nanoparticles (AgNPs) synthesized from agro-industrial byproducts were used as nanopriming agents for diploid (Riverside) and triploid (Maxima) watermelon seeds. Internalization of nanomaterials was confirmed by neutron activation analysis, transmission electron microscopy, and gas chromatography-mass spectrometry. The seedling emergence rate at 14 days after sowing was significantly higher in AgNP-treated triploid seeds compared to other treatments. Soluble sugar (glucose and fructose) contents were enhanced during germination in the AgNP-treated seeds at 96 h. Seedlings grown in the greenhouse were transplanted at four locations in Texas: Edinburg, Pecos, Grapeland, and Snook in 2017. At Snook, higher yield 31.6% and 35.6% compared to control were observed in AgNP-treated Riverside and Maxima watermelons, respectively. To validate the first-year results, treated and untreated seeds of both cultivars were sown in Weslaco, Texas in 2018. While seed emegence and stand establishments were enhanced by seed priming, total phenolics radical-scavenging activities, and macro- and microelements in the watermelon fruits were not significantly different from the control. The results of the present study demonstracted that seed priming with AgNPs can enhance seed germination, growth, and yield while maintaining fruit quality through an eco-friendly and sustainable nanotechnological approach.

## Introduction

Rapid and uniform seed germination is important for adequate crop establishment to ensure economic sustainability and efficient use of production resources in commercial agriculture^1^. This situation is particularly evident in high-value specialty crops such as watermelons where demand and production of seedless (triploid) varieties has become very popular compared to traditional seeded (diploid) varieties^2^. However, seedless (triploid) varieties have several production limitations, including low seed germination rates compared with diploid varieties, and generally lower stand establishment rates as a result of seedling sensitivity to environmental stresses. Seed characteristics partly account for these limitations. Triploid watermelon seeds are smaller in size, which has been associated with a limited amount of reserves to support germination and seedling growth. Significantly smaller lipid bodies and lower starch levels have been reported in triploid compared to the diploid seeds and these observations were correlated with significantly lower average germination rates for triploid seeds^3,4^. Besides size, other seed characteristics such as their thick seed coat, weak embryos, dense endotesta layers, and strong adherence of the seed coat to the cotyledon^5^ have also been noted as being significantly different from those of seeded varieties. Triploid seeds also tend to have an airspace surrounding the embryo, which may contribute to moisture deficit sensitivity^5^. It is possible that these seed characteristics have implications for water imbibition and onset of germination.

Semi-permeable layers in the seed coat of many crop species allow gas exchange and water uptake while restricting solute leakage. Watermelon seed coats have a thick aniline blue-staining layer^6^, which may affect water permeability and consequently, seed germination. Thicker seed coats in triploid watermelon varieties could further lead to oxygen deprivation, resulting in poor germination, inconsistent emergence, and poor stand establishment^5^. To improve seed coat permeability to water and oxygen, treatments such as seed coat removal, scarification, and seed nicking have been investigated with varying degrees of success for enhancing seed germination and seedling vigor. However, seedling vigor of triploid seeds is still lower than that of diploids^7^. Therefore, to improve seed germination and seedling vigor, novel seed priming techniques are needed.

Seed priming prior to sowing is a promising strategy to provide value-added solutions that enhance the yield and quality potential of high-value crops. Priming has been shown to result in higher emergence rates, vigorous seedling growth, and faster stand establishment rates. Priming induces an increase in the activity of enzymes such as amylases, proteases, and lipases that break down macromolecules for growth and development of the embryo. Priming also alleviates stress at the germination stage and ultimately results in higher rates of seedling emergence and successful seedling establishment^8,9^. These biological effects ultimately benefit farmers because they reduce the time, expense of re-seeding, additional irrigation, fertilization, and weed management on weak plants.

More recently, nanotechnology has emerged as an advanced seed priming technology for smart agriculture. Important and unique features of nanoparticles, such as their surface to mass ratio, which is much larger than that of other particles and materials, allows them to efficiently enhance catalysis, as well as to adsorb and deliver substances of interest^10^. Nanoparticles derived from metals or their compounds have been developed and used as carriers for biological systems. Our group has demonstrated significantly improved germination in diploid and triploid watermelon varieties with iron nanoparticle priming compared to the unprimed group^11^. This is probably due to the fact that nano-priming has the added advantage of being able to trigger certain metabolic processes that are normally activated during the early phase of germination. Consequently, nano-priming enhances the rate of emergence and subsequent growth, yield, and quality of the crop.

Synthesis of plant-based nanoparticles is a further refinement of nanotechnology that uses sustainable manufacturing processes to produce safe and innocuous nanoscale biomaterials for agricultural applications. Agro-industrial byproducts from turmeric and onions were used in this study to develop value-added nanomaterials that are environmentally benign and economically viable for large-scale production. Turmeric oil extracted from curcumin-removed turmeric oleoresin was used for the formulation of turmeric oil nanoemulsion (TNE). For preparing silver nanoparticles (AgNPs), onion peel extract was used as a reducing agent. Silver-based ‘green’ nanoparticles have the added protective advantage because silver has proven anti-bactericidal and anti-fungicidal properties^12^.

Based on the unique physical and chemical properties of nanoparticles and the aforementioned benefits as a delivery mechanism in biological systems, we hypothesized that seed priming with plant-based nanoparticles would improve germination/emergence rates, seedling vigor, and other growth parameters of triploid watermelons. Accordingly, this study investigated the effects of seed priming with green nanoparticles on various aspects of watermelon production, from seed germination to fruit yield and postharvest nutritional quality. The study also involved multi-location field investigations to assess if the nanoparticle-mediated benefits are region-specific. To the best of the authors’ knowledge, this is the first study to report the effects of nanopriming on seed germination, growth, yield, and nutritional qualities of watermelon.

## Materials and Methods

### Chemicals and materials

Silver nitrate (AgNO_3_) and surfactants polysorbate (Tween 20) and sorbitan monolaurate (Span 20) required for preparing AgNPs and TNE, respectively, were procured from Sigma-Aldrich (Sigma-Aldrich Chemical Co, St. Louis, MO, USA). The curcumin-removed turmeric oleoresin (CRTO) was obtained from Sami Labs Limited (Bangalore, India). Two hybrid varieties of watermelon seeds, Riverside (diploid) and Maxima (triploid) were obtained from Origene America Inc. (Alamo, TX, USA).

### Formulation of nanoemulsions using turmeric oil

Turmeric oil nanoemulsion formation was carried out using a low energy method based on a spontaneous emulsification procedure^13^ with minor modifications. The lipophilic phase was prepared by adding 125 mg of Span 20 in 25 mL of nanopure water followed by two hours of stirring on a benchtop magnetic stirrer (VWR International, Missouri City, TX, USA) at 1500 rpm. Similarly, the aqueous phase was prepared by adding 450 mg of Tween 20 in 75 mL of nanopure water with agitation by a magnetic stirrer for two hours. In this spontaneous emulsification method, 2 mL turmeric oil and lipophilic surfactant (25 mL) were mixed together and then the mixture was poured into 75 mL of aqueous phase while continuously stirring the system with the magnetic stirrer at ambient temperature and kept under vigorous stirring overnight to prepare the TNE.

### Green synthesis of silver nanoparticles

AgNPs were prepared using onion peel water extract as a reducing agent. Onion peel (25 g) was boiled for 10 min in 200 mL of nanopure water. The extract was filtered through Whatman filter paper grade 1 followed by a cellulose filter for the synthesis of AgNPs. AgNPs were prepared by adding 0.5 mL of freshly prepared onion extract to 10 mL of 0.01 M AgNO_3_ solution at 80 °C with continuous stirring for 5 minutes. A reddish dark brown color was observed after addition of onion peel extract indicating the formation of AgNPs.

### Characterization of nanoemulsions and nanoparticles

The prepared nanomaterials were characterized by using a dynamic light scattering (DLS) technique (Malvern Zetasizer Nano-ZS model, Malvern, UK), according to our published protocol^14^. Ultraviolet-visible spectroscopy (UV-2900 Hitachi spectrophotometer) was used for optical absorption measurements of prepared AgNPs.

Similarly, the morphology and size of the AgNPs was captured by transmission electron microscopy (TEM) at 100 kV. X-ray diffraction (XRD) was used to fully understand and confirm the crystal structure and crystalline size of the AgNPs. The powder forms of AgNPs were subjected to X-ray diffraction analysis (D8 Powder Eco) at 40 kV and 25 mA with Cu Kα radiation. The scan 2θ range was 20–80°.

### Seed treatments

Two varieties of watermelon seeds, Riverside and Maxima, were primed with TNE and AgNPs in 2017 and 2018. One-part TNE was diluted with ten parts of nanopure water to make the priming solution. A 2.5% suspension of synthesized AgNPs was used, which contains 31.3 ppm of Ag. The concentrations of nanopriming agents were selected based on preliminary experiments. The unprimed seeds were used as control. In third year 2019, along with unprimed seeds, AgNO_3_ primed (same concentration as AgNPs), and hydroprimed seeds were also added as controls. Seeds were immersed in priming media for 12 h, in the dark, at room temperature, and the proportion of seed weight to priming solution was 1:5 g/mL. Seeds were dried at ambient temperature after rinsing 2–3 times with nanopure water.

### Quantification of internalized nanomaterials in watermelon seeds

#### TNE in watermelon seeds

TNE-treated seeds were washed with water repeatedly to remove the adhering emulsion, then the seeds were crushed. A known amount (100 mg) of TNE-treated sample was extracted with 0.6 mL hexane twice and the pooled extract was filtered and used for GC-MS analysis for the identification and quantification of the major volatile compounds. The Thermo Finnegan gas chromatograph (Thermo Fisher Scientific, Inc., San Jose, CA, USA) coupled with an electron ionization (EI) source with a Dual-Stage Quadrupole (DSQ II) mass spectrometer (Thermo Scientific, Austin, TX, USA) was used for volatile analysis. Chromatographic separation was carried out on a polar phase column Rtx-Wax of 30 m × 0.25 mm i.d. 0.25 um film thickness) (Restek Corporation, Bellefonte, PA, USA). Helium was used as a carrier gas with a flow rate of 1 mL/min and sample volume 2 μL was injected into the GC injector at 225 °C. The initial oven temperature was maintained at 50 °C, then increased to 230 °C at a rate of 10 °C/min, held for 5 min, with a total run time of 23 min. Electron impact (EI) data from m/z 40 to 400 were acquired at a scanning speed of 16.67 scans/sec. The temperatures of ion source and mass transfer line were maintained at 285 °C and 280 °C, respectively. The data were processed using Xcalibur software (v. 2.0.7., Thermo-Fisher Scientific, San Jose, CA, USA).

#### AgNPs in watermelon seeds

Mass fractions of Ag were determined by comparative instrumental neutron activation analysis (INAA)^15^. In order to identity and quantify Ag in watermelon seed samples, three sets of control and AgNP-treated seeds were irradiated for 14 h using 1-MW TRIGA reactor. Following an 11-d decay interval, gamma-ray spectra were acquired for 1 h each using an HPGe detector. These spectra were used to quantify Ag using the mean values of three gamma-ray peaks (658, 885, and 1384 keV) from the radioisotope ^110m^Ag (t_1/2_ = 249.76 d). The data reduction was conducted using the NAA software package (OpenVMS alpha processor-based Genie-ESP software) from Canberra Industries (Meriden, CT, USA).

### Observation of seed ultrastructure

Randomly selected AgNP-primed seeds were dissected and pre-fixed using Trump’s fixative and washed with Trump’s buffer. Post-fixation with 1% osmium tetroxide was done. The specimen was infiltrated after dehydration through a series of acetone concentrations, then it was fixed in Spurr resin, and polymerized at 65 °C for 24 h. Leica Ultracut UCT ultra-microtome was used to prepare Ultrathin sections. TEM images were obtained using a JEOL 1200Ex (JEOL, Tokyo, Japan) TEM at an accelerating voltage of 100 kV.

### Assessment of germination of watermelon seeds

The incubator study was conducted in darkness at 30 °C using Riverside (diploid) and Maxima (triploid) seeds with three treatments: control (unprimed seeds), TNE- and AgNP-treated seeds. Uniform size seeds were placed in 100 ×15 mm Petri dishes with 25 seeds per dish and each test was replicated three times. Radicle emergence to 2 mm or more was scored as germination and was recorded at 24 h intervals for ten days. Two mL of distilled water was added in alternate days to all petri dishes to prevent drying. The number of germinated seeds was counted every day. Final germination percentage (FGP), time to 50% germination (T50), germination rate (GR), and mean germination time (MGT) were considered to study the effect of nanopriming on the germination of watermelon seeds and calculated using the published equation^16^. This experiment was repeated in the second year, 2018. In the third year 2019, the incubator study was conducted with five treatments: unprimed, hydroprimed, TNE-, AgNO_3_- and AgNP-treated seeds. Based on the International Seed Trade Association (ISTA) guidelines, 400 seeds were tested for each treatment. For each treatment, 16 petri dishes containing 25 seeds in each replicate were tested.

### Analysis of sugar by HPLC

Germinated seed samples from each group were collected in 24-h intervals starting from the samples just after priming (i.e., 0 h) to 96 h. Seeds were ground in liquid nitrogen with a mortar and pestle and freeze-dried. Aliquots of 100 mg freeze-dried seed samples were transferred into 1.5 mL centrifuge tubes and 600 μL of nanopure water was added. All samples were vortexed for 30 s, sonicated for 15 minutes, and centrifuged (10,000 × g, 10 min). The supernatant was separated, and the residue was reextracted with 400 μL of nanopure water. Both extracts were combined and used for the HPLC analysis according to our published method^17^.

### Emergence study

A seedling was considered emerged when the cotyledons were completely raised above the media. Emergence tests were conducted using Riverside (diploid) and Maxima (triploid) seeds with a total of three treatments, i.e. control (unprimed seeds), TNE, and AgNPs. For emergence tests, seeds with different treatments were sown at the greenhouse. Twenty seeds of each treatment were individually sown in 200-cell plastic trays containing a commercial growing medium with three replications. Various results have been obtained in previous studies relating cucurbit seed orientation to emergence^18^. In this experiment, the seeds were oriented with the radicle end up at a 90° angle to reduce the seed coat adhered to cotyledons. Trays were thoroughly moistened and moisture levels were maintained throughout the experiment. Stem length and diameter were recorded at 21 days after sowing and the same seedlings were used as watermelon transplants for the field experiment. The entire emergence experiment in the greenhouse was repeated in the second year, 2018. In third year 2019, emergence study was conducted with total five treatments: unprimed, hydroprimed, TNE-, AgNO_3_- and AgNP-treated seeds. All the other parameters remained the same.

### Measurement of chlorophyll in watermelon seedlings

The interaction between Chl molecules and NPs was investigated in watermelon seedlings at 21 days after sowing (DAS) to understand the potential toxicity of seed priming with the TNE and AgNPs. Leaf samples were ground in liquid nitrogen with mortar and pestle and extracted in dark conditions. One mL of acetone was added to 300 mg of leaf sample. Each sample tube was vortexed (30 s), sonicated (15 min at 4 °C), and centrifuged for 10 minutes. After, decanting and filtering the extract, the residue was re-extracted with 0.5 mL of acetone. Both extracts were combined and filtered through 0.45-micron filters then used for the HPLC analysis according to our published method^19^. To understand the potential toxicity of seed priming with AgNPs in comparison with its bulk counterpart AgNO_3_ at the same concentration, chlorophyll was measured in the leaf tissue of 14-day-old watermelon seedlings collected from the third-year emergence study.

### Transplanting watermelon seedlings

Field experiments were conducted to evaluate greenhouse results in field conditions. Growing location and other environmental factors can significantly affect the growth and yield of watermelon. Watermelons seedlings (5–7 weeks old) were transplanted at four different locations in Texas; Edinburg (26 °18′15′′N 98 °9′50′′W), Snook (30 °29′25′′N 96 °28′11′′W), Pecos (31 °24′56′′N 103 °30′0′′W), and Grapeland (31 °29′30′′N 95 °28′49′′W) in the grower’s field during the summer season of 2016/17. The four distinct research locations in Texas have unique conditions, which represent different climatic conditions, soil fertility, and soil types. A field trial was conducted with a randomized complete block design with four replications and three treatments, unprimed, TNE, and AgNPs for both varieties (Riverside and Maxima). Field plots were prepared by forming raised beds covered with/without black plastic mulch and drip tape in the bed. A plant-to-plant distance of 3.5 feet was maintained within the row with a planting density of six plants per plot. In the case of triploid (Maxima) plots, two pollen-producing plants (pollinizers) per plot were planted making a 1:3 ratio of diploid and triploid plants. Pollinizers were interplanted in the same row as the triploid variety. The soil was collected from all four locations for routine analysis.

Moreover, to observe the direct seeding effect and to validate the result of the first year, another field trial was conducted in Texas A&M AgriLife Research and Extension Center at Weslaco (26 °15’N, 97 °98’W) where treated (TNE and AgNPs) and unprimed seeds were directly sown in the field. This project was conducted during the summer season of 2017–18 with a total of six replications and planting density of five plants per plot. In the case of triploid (Maxima) plot, one pollinizer per plot was planted making a 1:4 ratio of diploid and triploid plants in the second year.

### Assessment of growth and fruit yield of watermelons

Length of the main vine and stem diameter were recorded for 40-day-old vines in all five locations. The length was measured from the base of the plants to the tip of the main vine and the stem diameter was recorded at the base of the plant. The fields were harvested when more than 90% of the fruits were ripe. Fruit was considered to be ripe by looking for a dried tendril nearest the fruit, a dull sound of the fruit when thumped and a light-colored ground spot, as is normally done for watermelon maturity determination.

In the first year, out of four research locations, fields at Grapeland and Snook were selected for a yield study as shown in the map (Supplementary Information, Fig. S1). Fruit was harvested three times at Grapeland and Snook. In each harvest, the fruit was counted and weighed for each treatment. Total fruit weight for the three harvests combined was calculated. During the second year, owing to the bad weather conditions (heavy storms and rainfall in June that led to flooding in the watermelon field after the first harvest), a single harvest was done in Weslaco. Weather data are given in Supplementary Information, Fig. S2.

### Determination of total phenolics and radical scavenging activity

Total phenolics and radical scavenging activity of watermelon fruit harvested from all locations were quantified using our previously published method with slight modifications^20^. Fresh watermelon fruit samples (15 g) were extracted twice with 15 mL methanol and the extracts were pooled. All standards and samples were pipetted in triplicate into 96-well plates. The absorbance was measured using a Synergy HT Multi-Mode Microplate Reader (BioTek, Instruments, Winooski, VT, USA) at 760 nm and 515 nm for total phenolics and DPPH assays, respectively. The total phenolics were expressed as mM Trolox equivalents per gram of fresh weight of samples, and radical-scavenging activity was expressed as mg of ascorbic acid equivalents per gram dry weight.

### Mineral contents

Nano-treated and untreated watermelon pulps harvested from all the locations in the first year (Snook, Grapeland, Edinburg, and Pecos) and second year (Weslaco) were freeze-dried. Samples from diploid and triploid watermelon cultivars were submitted to the Soil, Water and Forage Testing Laboratory, Texas A&M University, College Station, Texas. The macro-elements nitrogen (N), phosphorus (P), sulphur (S), potassium (K), calcium (Ca), sodium (Na), and magnesium (Mg) were measured along with the microelements iron (Fe), copper (Cu), manganese (Mn), boron (B), and zinc (Zn). Nitrogen content was determined by the high-temperature combustion process. The inductive coupled plasma-atomic emission spectroscopy (ICP-AES) (Spectro Genesis, Deutschland, Germany) was used to estimate the other mineral (P, K, Ca, Mg, Na, S, Fe Cu, Mn, Zn, and B) contents.

### Statistical analysis

One-way analysis of variance (ANOVA) was performed using JMP software (JMP pro 14). Significant differences were tested using a general linear model and means were compared using Student’s *t*-test at 5% probability level (*p* ≤ 0.05). The results are expressed as means ± standard error of the mean. Microsoft Excel was used for data visualization and graphing.

## Results

### Synthesis and characterization of the TNE and AgNPs

Turmeric oil nanoemulsion was prepared using a low-energy method based on a spontaneous emulsification technique. The nanoemulsion was optically opaque, homogeneous, and physically stable. Dynamic light scattering technique was used to record the mean particle size of TNE which was 171.3** ±** 0.52 nm with PDI and ZP values of 0.25 and −1.23** ±** 0.16 mV, respectively (Supplementary Information, Fig. S3A).

In the case of AgNPs, a reddish dark brown color was observed when Ag^+^ ions are reduced to AgNPs after addition of onion peel extract indicating a reduction of Ag^+^ to Ag^o^. The mean particle sizes recorded for AgNPs was 141.3** ±** 0.78 nm with PDI and ZP values of 0.18** ±** 0.03 and −1.23** ±** 0.16 mV, respectively (Supplementary Information, Fig. S3B). The surface plasmon resonance (SPR) of AgNPs showed a peak centered near 410 nm in UV-vis spectra (Supplementary Information, Fig. S3C), confirming that the phytochemicals present in onion peel extract reduced the silver salt into AgNPs.

**Figure 1:**
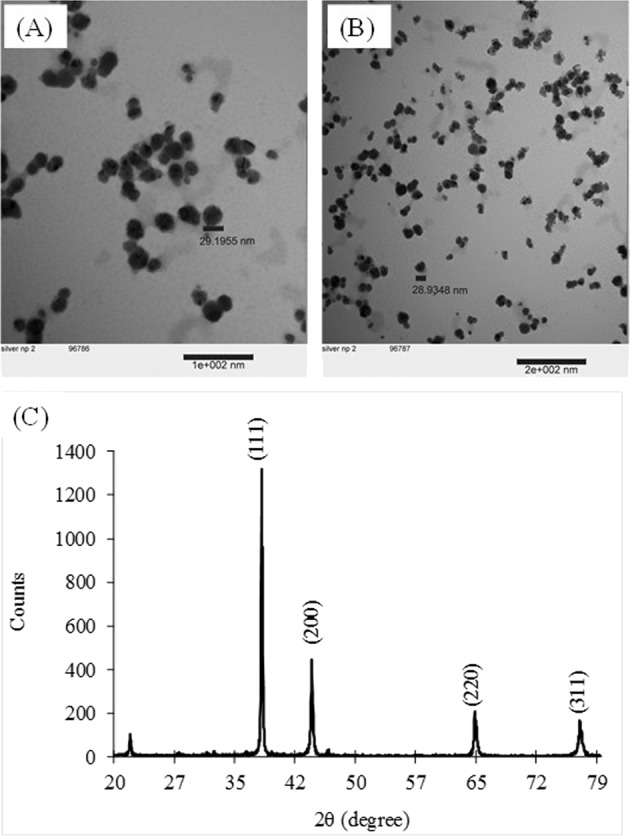
TEM images of silver nanoparticles showing the morphology of synthesized silver nanoparticles at (**A**) scale bar 100 nm (**B**) scale bar 200 nm, (**C**) X-ray diffraction pattern of silver nanoparticles synthesized using onion peel extract.

Transmission electron microcopy images (Fig. 1A,B) of AgNP samples showed typical spherical and ellipsoidal morphology with approximate particle diameter of 29 nm. Crystalline nature of synthesized AgNPs was confirmed using X-ray diffraction (XRD). Figure 1C displays four prominent diffraction peaks at 2θ value of 38.19°, 44.38°, 64.49°, and 77.45° indexed as (111), (200), (220), and (311) Miller indices, respectively, which are characteristic of face centered cubic (fcc) crystalline structure of metallic silver (JCPDS file No. 04–0783). The average nanocrystalline size of synthesized AgNPs was calculated using Debye-Scherrer’s equation and was found to be around 36.5 nm (Supplementary Information, Table S1).

### Enhancement of seed germination, emergence, and seedling growth rate by nanopriming

To examine the effect of nanopriming with AgNPs and TNE, we first assessed seed germination and seedling emergence in 2017 and 2018. The control unprimed treatment required significantly more days for 50% germination of Riverside and Maxima cultivars during the first and second years, respectively, as compared to AgNP treatment (Table 1). Conversely, seeds of Riverside variety treated with AgNPs had the lowest mean germination times (MGT) and the highest germination rates (GR) compared to the other treatments, showing significant differences in MGT and GR. TNE-treated seeds had significantly higher GR than control, unprimed seeds of Riverside and Maxima cultivars. However, there were no significant differences in the number of days required for 50% seed germination, final germination percentage (FGP), and the final emergence percentage (FEP) when seeds were treated with TNE compared to the unprimed watermelon seeds of both varieties during both planting seasons. No differences were observed in the FGP in both cultivars among the treated and untreated seeds. FEP was significantly higher in the AgNP-treated Maxima seeds. However, no significant difference in FEP was observed for Riverside seeds during both years. The increased FEP suggests that AgNP treatment is more effective at enhancing emergence in triploid cultivars as compared to diploids.

**Table 1:** Germination and emergence of nanoparticle-treated watermelon seeds in 2017, 2018, and 2019.

Year	Variety	Treatments	Days required for 50% germination	FGP (5 DAS)	MGT (days)	GR (seed/day)	FEP (14 DAS)
2017	Riverside	UP	2.33 ± 0.33^a^	100 ± 0.00^a^	2.37 ± 0.09^a^	0.42 ± 0.02^c^	96.66 ± 3.36^a^
		TNE	2.00 ± 0.00^a^	100 ± 0.00^a^	1.95 ± 0.08^b^	0.51 ± 0.02^b^	93.33 ± 1.68^a^
		AgNPs	1.66 ± 0.33^a^	100 ± 0.00^a^	1.60 ± 0.14^c^	0.63 ± 0.05^a^	96.66 ± 3.36^a^
	Maxima	UP	2.00 ± 0.00^a^	100 ± 0.00^a^	2.24 ± 0.07^a^	0.45 ± 0.01^c^	70.00 ± 2.91^ab^
		TNE	2.00 ± 0.00^a^	100 ± 0.00^a^	1.76 ± 0.04^b^	0.57 ± 0.01^b^	58.30 ± 7.32^b^
		AgNPs	1.33 ± 0.33^b^	100 ± 0.00^a^	1.52 ± 0.08^c^	0.66 ± 0.03^a^	80.00 ± 2.91^a^
2018	Riverside	UP	2.00 ± 0.00^a^	99 ± 0.67^a^	2.04 ± 0.01^a^	0.49 ± 0.00^c^	98.33 ± 1.67^a^
		TNE	2.00 ± 0.00^a^	99 ± 0.67^a^	1.74 ± 0.03^b^	0.57 ± 0.01^b^	98.33 ± 1.67^a^
		AgNPs	1.00 ± 0.00^b^	100 ± 0.00^a^	1.16 ± 0.02^c^	0.85 ± 0.02^a^	98.33 ± 1.67^a^
	Maxima	UP	2.00 ± 0.00^a^	94 ± 1.71^a^	2.12 ± 0.02^a^	0.47 ± 0.00^c^	58.73 ± 1.67^a^
		TNE	2.00 ± 0.00^a^	96 ± 1.79^a^	1.81 ± 0.03^b^	0.54 ± 0.01^b^	72.22 ± 7.22^ab^
		AgNPs	2.00 ± 0.00^a^	98 ± 0.89^a^	1.49 ± 0.05^c^	0.67 ± 0.02^a^	85.55 ± 7.22^a^
2019	Riverside	UP	2.00 ± 0.00^b^	94 ± 1.36^b^	2.26 ± 0.04^a^	0.44 ± 0.01^d^	90.47 ± 4.76^a^
		HP	1.93 ± 0.11^b^	94 ± 0.89^b^	1.82 ± 0.06^b^	0.56 ± 0.02^c^	90.47 ± 4.76^a^
		TNE	1.18 ± 0.10^c^	96 ± 1.02^ab^	1.37 ± 0.06^c^	0.75 ± 0.03^b^	95.23 ± 4.76^a^
		AgNO_3_	3.00 ± 0.00^a^	79 ± 1.74^c^	2.59 ± 0.25^a^	0.30 ± 0.03^e^	66.66 ± 4.76^b^
		AgNPs	1.00 ± 0.00^c^	98 ± 0.72^a^	1.17 ± 0.02^c^	0.86 ± 0.02^a^	95.23 ± 4.76^a^
	Maxima	UP	2.00 ± 0.00^a^	94 ± 1.41^a^	2.30 ± 0.05^b^	0.44 ± 0.01^c^	52.38 ± 4.76^b^
		HP	1.63 ± 0.13^b^	95 ± 0.70^a^	1.82 ± 0.11^bc^	0.57 ± 0.02^b^	61.90 ± 4.76^ab^
		TNE	1.19 ± 0.10^c^	95 ± 0.40^a^	1.74 ± 0.13^bc^	0.65 ± 0.07^b^	66.66 ± 4.76^ab^
		AgNO_3_	—	16 ± 2.55^b^	3.35 ± 0.59^a^	0.27 ± 0.04^d^	0.00 ± 0.00^c^
		AgNPs	1.00 ± 0.00^c^	98 ± 0.73^a^	1.23 ± 0.05^c^	0.84 ± 0.04^a^	76.19 ± 4.76^a^

Data from 2017 and 2018 are presented as mean ± SEM from three replicates containing 25 seeds of each treatment. Data from 2019 are presented as the mean of 16 replicates containing 25 seeds each ± SEM. Means denoted by the different letters are significantly different at p ≤ 0.05.

UP: Unprimed, HP: Hydroprimed, TNE: Turmeric oil nanoemulsion, AgNO_3_: Silver nitrate, AgNPs: Silver nanoparticles, FGP: Final germination percentage, MGT: Mean germination time, GR: Germination rate, FEP: Final emergence percentage, DAS: Days after sowing.

In third year 2019, the incubator and greenhouse studies were conducted with five treatments: unprimed, hydroprimed, TNE-, AgNO_3_- and AgNP-treated seeds. The main objective of this experiment was to compare the effect of seed treatment with the same concentration of AgNPs and their bulk counterpart, AgNO_3_ solution. When the effect of AgNO_3_ and AgNPs was compared in the repeated germination study, we found that AgNO_3_ significantly reduced germination in both cultivars (Table 1). In AgNO_3_-treated watermelon seeds, the FGP was 79% for Riverside and 16% for Maxima, whereas FGP was significantly higher in AgNP-treated seeds (98% for both cultivars).

We also conducted a greenhouse study in the third year to compare the FEP between AgNO_3_ and AgNP treatments. The FEP was 67% and 95% in Riverside watermelon using AgNO_3_ and AgNPs, respectively. In Maxima, severe toxicity of AgNO_3_ was observed, resulting in no emergence. This indicates that the toxicity of bulk metal particles is cultivar and ploidy dependent (Maxima = 3n; Riverside = 2n). Similarly, hydropriming was included as the other control in the third-year incubator and greenhouse study. Although the FGP and FEP were similar in the unprimed and hydroprimed seeds, significantly enhanced growth rate and reduced mean germination time was observed in the hydroprimed seeds as compared to the unprimed control. However, TNE and AgNPs had significantly higher growth rate and reduced mean germination time as compared to the hydroprimed seeds of both cultivars.

### Influence of seed germination on primary metabolites

**Figure 2:**
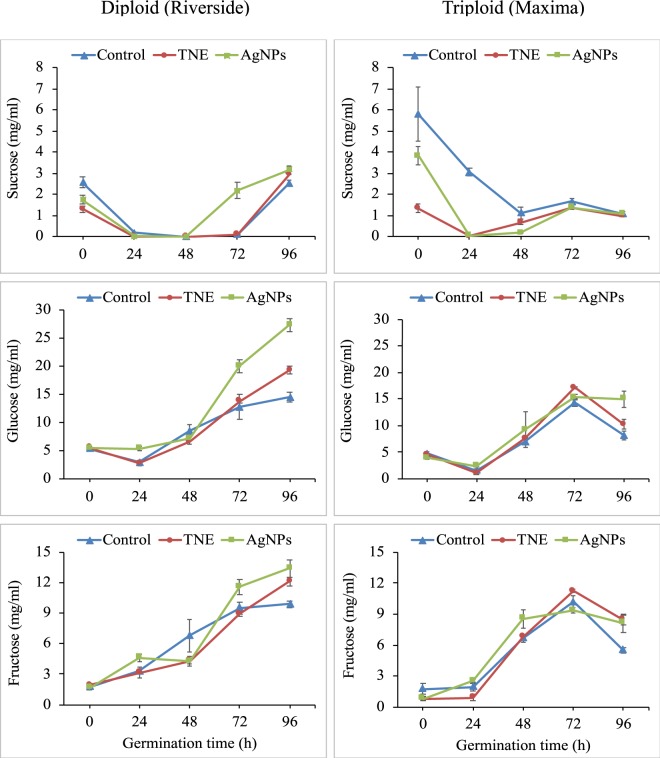
Effect of nanoparticle treatment on diploid (Riverside) and triploid (Maxima) watermelon seeds up to 96 h. Rapid degradation of polysaccharides and disaccharides in nanoparticle-treated samples shown at different time points. Control is unprimed, TNE is turmeric oil nanoemulsion and AgNPs is silver nanoparticles. Values are mean ± SEM (n = 6).

During germination, disaccharides (sucrose) are converted to monosaccharides (glucose and fructose) to fuel development of the germinating seedling (Fig. 2); therefore, we next assessed the effect of nanopriming on this key germination process. An initial reduction in sucrose content of treated and untreated diploid seed was observed at 24 h after priming, suggesting that carbohydrate mobilization and metabolism are highly active in that stage. In the case of triploid seeds, the sucrose level was reduced in nanoprimed seeds at 24 h and in control seeds at 48 h. This shows the influence of nanopriming on seed germination. Glucose and fructose levels peaked at 96 h after priming in the diploid cultivar while in the triploid, glucose and fructose levels declined after 72 h. Interestingly, at 96 h after priming, the glucose and fructose levels were relatively higher in nanomaterial-treated seeds compared to the unprimed control of both varieties.

### Effect of nanopriming on photosynthetic pigments

**Figure 3:**
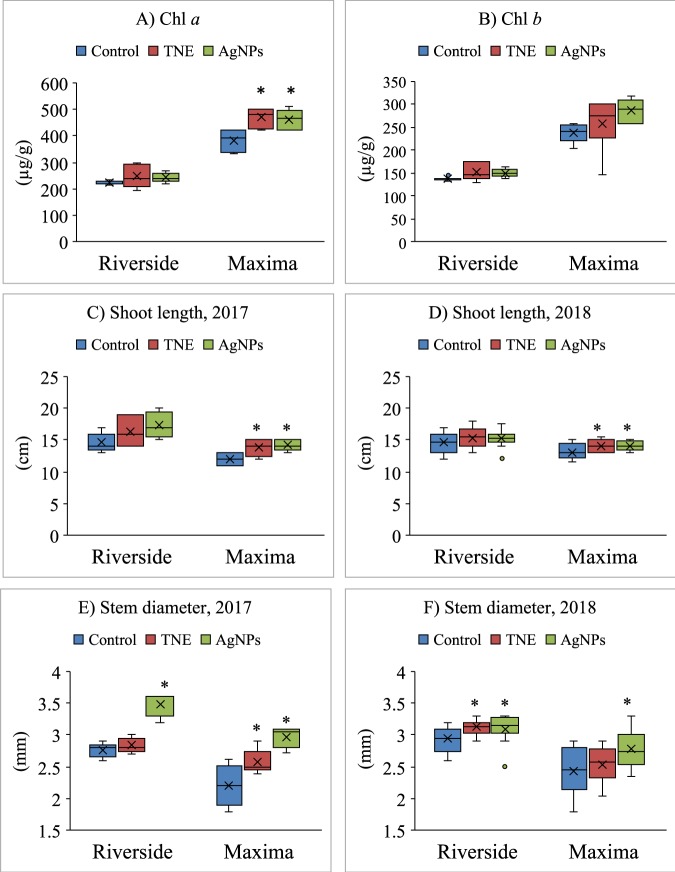
Effect of nanoparticle treatments on chlorophyll content, stem diameter and shoot length in watermelon seedlings at 21 days after transplanting. (**A**) Chlorophyll *a* (Chl *a*) and (**B**) chlorophyll *b* (Chl *b*) content in Riverside and Maxima watermelon seedlings at 21 days after sowing (DAS). Boxplots show the shoot length of watermelon grown in (**C**) first year, 2017 and (**D**) second year, 2018; stem diameter of watermelon grown in (**E**) first year, 2017 and (**F**) second year, 2018. Control is unprimed, TNE is turmeric oil nanoemulsion and AgNPs is silver nanoparticle-treated watermelon seedlings. *Denotes statistical differences between treatments at the 5% level according to t test and ‘×’ denotes mean.

If nanopriming caused toxicity in the developing seedlings, we might see a decrease in photosynthetic pigments; by contrast, if nanopriming improved seedling vigor, we might see an increase in photosynthetic pigments compared with control. Effects of seed priming with TNE and AgNPs on seedling leaf chlorophyll (chl) *a* and *b* contents were assessed at 21 DAS. Seedlings of nano-primed seeds had elevated or similar levels of chl *a* and chl *b* compared to control (Fig. 3A,B). Nanopriming had a significant influence on chl *a* content of the triploid watermelon seedlings. Although there were no significant differences, both nanotreatments enhanced the chl *a* and chl *b* contents of diploid watermelon seedlings. These results indicate that seed treatment with TNE and AgNPs had no discernable negative effects on watermelon seedling growth.

### Growth of watermelon transplants

To test whether the effects we observed on the seedlings carried over into improved growth later in development, we next examine growth in transplanted plants. To this end, shoot length and stem diameter were recorded at 21 DAS watermelon seeds. Maxima seedlings from both nano-priming treatments had significantly longer shoots compared to the control. Although treated seedlings had longer shoots than the control, there was no significant difference in the Riverside variety. This trend was similar in both planting years (Fig. 3C,D). AgNP-treated seedlings had significantly larger stem diameters (Fig. 3E,F) in both Riverside and Maxima cultivars as compared to control during both planting years.

### Nanopriming to enhance growth parameters of watermelon vine

**Figure 4:**
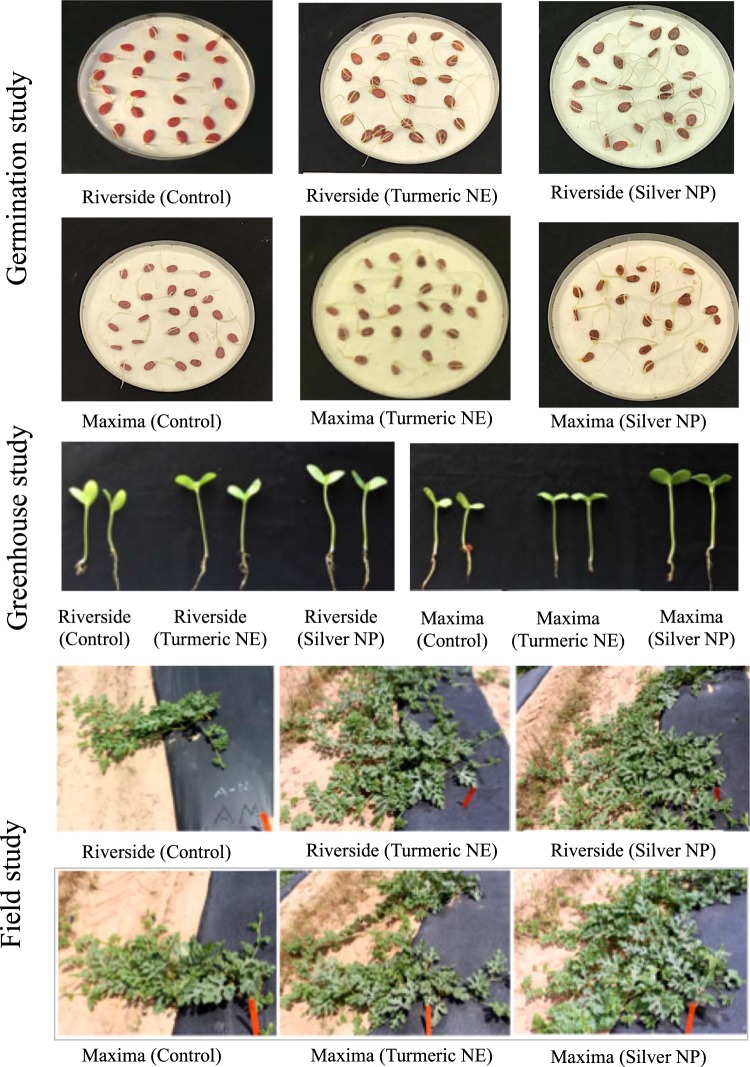
Effect of seed priming with turmeric nanoemulsion and silver nanoparticles on the germination, seedling growth and vine development of diploid (Riverside) and triploid (Maxima) watermelon varieties, compared with unprimed control.

**Figure 5:**
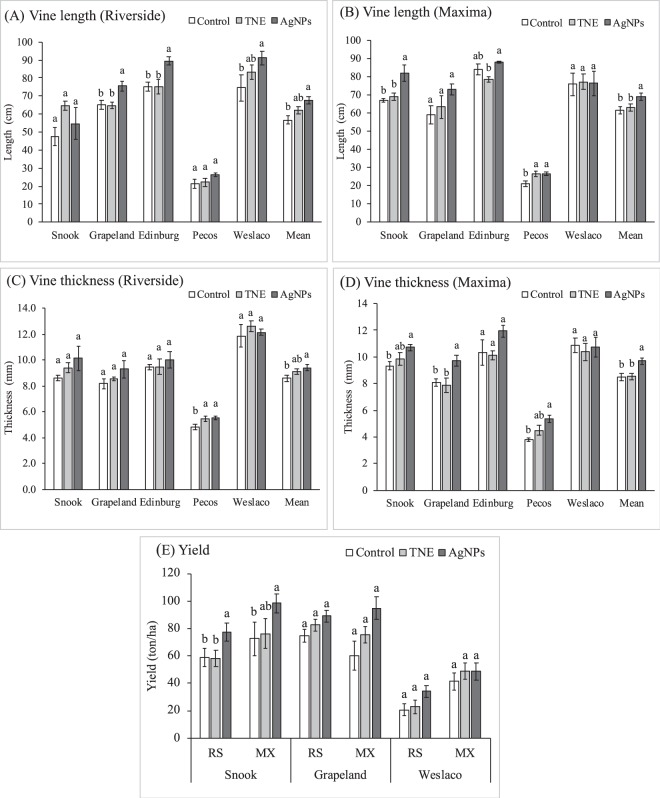
Effects of nanoparticles on the growth parameters at 40-day-old watermelon plants and yield. (**A**) Vine length (cm) of Riverside, (**B**) vine length (cm) of Maxima, (**C**) vine thickness (mm) of Riverside, and (**D**) vine thickness (mm) of Maxima watermelon plants grown in five locations of Texas (Snook, Grapeland, Edinburg, Pecos, and Weslaco). (**E**) Effect of nanopriming on yield of watermelon grown in three locations in Texas (Snook, Grapeland and Weslaco). Watermelons were harvested three times during the first year at Snook and Grapeland. Due to bad weather conditions, a single harvesting was done in Weslaco. Control is unprimed, TNE is turmeric oil nanoemulsion and AgNPs is silver nanoparticles treated watermelon. Different letters denote significant difference (*p* ≤ 0.05) between treatments. Data represent mean ± SEM.

To test these effects in field conditions that are relevant to agricultural production, we transplanted watermelon seedlings into growers’ fields in four different locations in Texas: Edinburg, Grapeland, Snook, and Pecos (Supplementary Information, Fig. S1). In the field study also, nanoparticle-treated seeds grew very well as compared to the unprimed controls (Fig. 4). To study the effect of nanopriming on growth, we measured vine length and vine thickness at 40 days after transplanting (DAT) at all the growing locations. The length of the main vine was significantly higher in the AgNP-treated plants as compared to the other treatments in both Maxima and Riverside cultivars grown in Edinburg (Fig. 5A,B). In Grapeland, Snook, and Pecos, vine length was significantly higher in the AgNP-treated plants as compared to control only in the Maxima cultivar. Effect of the TNE treatment was not significantly different from the control for the vine length of Riverside in all the locations, but TNE treatment enhanced growth of the Maxima cultivar grown in Pecos.

Stem diameter showed a significant difference in Maxima but not in Riverside cultivar grown in Grapeland and Snook (Fig. 5C,D). AgNP-treated plants had thicker stems as compared to other treatments in the Maxima cultivar. No significant differences were observed in the stem diameter between the treatments in Edinburg. In both cultivars grown at Pecos, stem diameter was significantly higher in NP-treated watermelon plants as compared to the control. During the second year at Weslaco, vine length and thickness were recorded at 40 DAS and AgNP-treated Riverside watermelons had significantly longer vines, but no difference was observed in the Maxima cultivar. Similarly, no significant differences were observed in the stem diameter between the treatments in Weslaco.

Watermelons grown at Pecos had poor growth performance at 40 DAT as compared to the other locations. This effect on growth parameters among the five locations (Supplementary Information, Fig. S1) could be due to environmental factors such as weather conditions (Supplementary Information, Fig. S2), soil type (Supplementary Information, Table S2), and water availability. Pecos has a semiarid to desert climate with hot summers. The low rainfall of this region is reflected in the slow growth of the watermelon vines compared to the other locations.

### Effect of nanopriming on watermelon yield

Two nanopriming-based treatments (TNE and AgNPs) were used for this study and compared with the control. Out of four locations, the two fields nearest the university, Grapeland (90 miles) and Snook (14 miles), were selected for yield measurements in the first year. These two locations were selected based on the feasibility of conducting multiple harvests and intensive management. In order to observe the direct seeding effect and to validate the results of the first year, another field trial was conducted in Texas A&M AgriLife Research and Extension Center at Weslaco, which is the best production area for watermelon of the available sites in Texas. In both varieties, AgNP-treated watermelons had a significantly higher yield (tons/ha) than the other two treatments (control and TNE) in Snook (Fig. 5E). Higher yield (31.6%) was observed in AgNP-treated plants compared to control in Riverside watermelons at Snook. Similarly, for Maxima, AgNP treatment increased yield by 35.6% at Snook compared to the control. No significant difference in yield was observed between control and TNE.

In Grapeland and Weslaco, for both varieties, all three treatments showed no statistically significant differences in yield. However, AgNP-treated watermelons had a higher yield as compared to the control in both locations.

### Internalization studies

To understand how nanopriming could affect the growth of watermelon plants, we measured the internalization of major active compounds present in TNE by gas chromatography-mass spectrometry (GC-MS; Supplementary Information, Table S3). Ar-turmerone is the major compound present in the TNE, and it was used as a marker to test internalization into the watermelon seeds. The levels of ar-turmerone internalized in TNE-treated Riverside and Maxima seeds were 2250.18 μg/g and 2422.46 μg/g, respectively.

**Figure 6:**
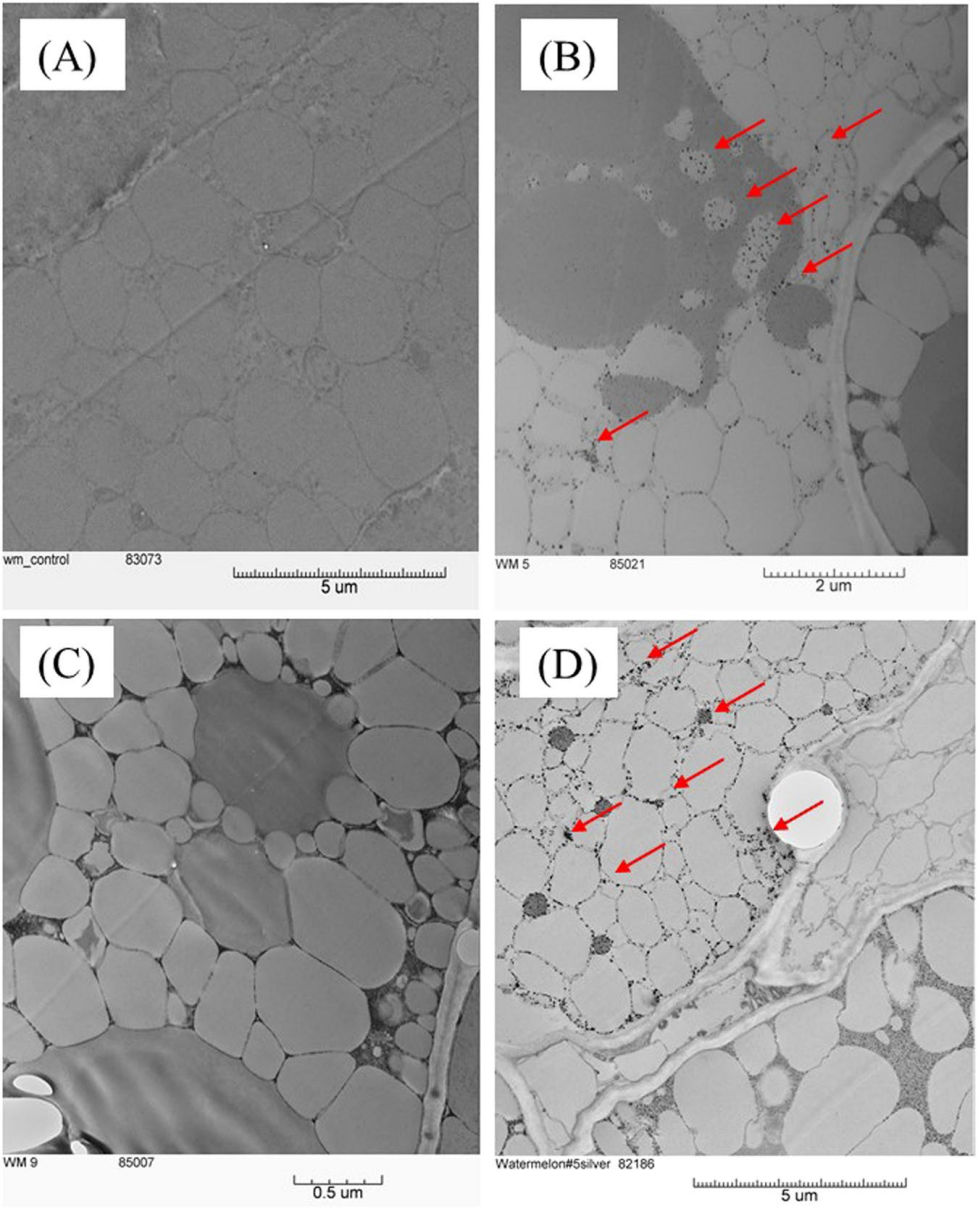
Transmission electron microscopy (TEM) images of internalization of silver nanoparticles in the (**A**) unprimed triploid (Maxima) seed, (**B**) AgNP-primed Maxima seed (**C**) unprimed Riverside seed and (**D**) AgNP-primed Riverside seed. Arrows indicate regions where AgNPs (dark bead like structures) have accumulated in treated seeds.

Internalization of Ag was determined by INAA (Supplementary Information, Table S3). Ag was detected in the treated seeds of both varieties, while in the unprimed seeds, the Ag level was below the detection limit (detection limit of Ag by INAA is 40 ng/g). The concentration of Ag in the AgNP priming solution was 31.3 μg/mL. After 12 h priming, the concentrations of Ag absorbed by the Riverside and Maxima seeds were 20.86 and 15.63 μg/g fresh weight, respectively. This result suggested that Ag^+^ ions released from AgNPs penetrate the seed coat into seed tissues. The findings were further confirmed by TEM images of watermelon seeds (Fig. 6) which showed that AgNPs were found inside seed embryos in the treated seeds and no particles were observed in the control, unprimed watermelon seeds.

### Effect of nano-treatments on total phenolics, radical scavenging activity, and elemental composition in watermelon fruit

**Figure 7:**
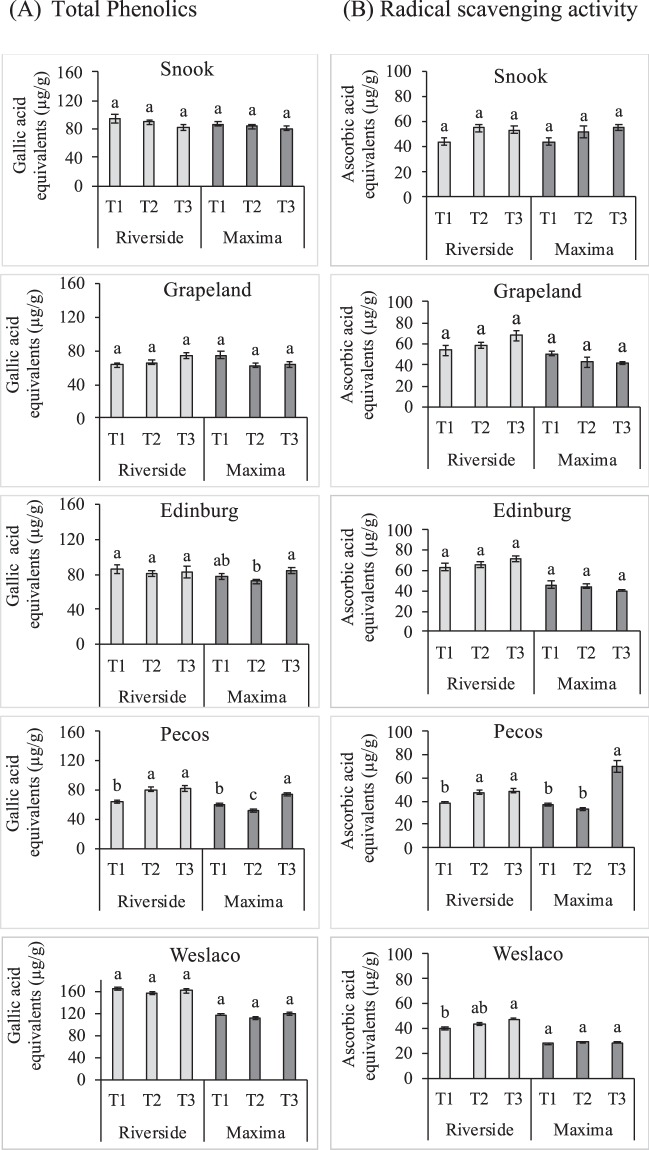
Nanopriming effects on (**A**) Total Phenolics and (**B**) Radical scavenging activity of watermelon fruit; Riverside and Maxima by DPPH method. T1: unprimed, T2: turmeric oil nanoemulsion and T3: silver nanoparticles. Data are presented as means ± SEM (n = 12). Means denoted by the different letters are significantly different at (*p* ≤ 0.05).

Phenolic compounds have high antioxidant activities and free radical scavenging capacity, and inhibit the enzymes responsible for reactive oxygen species (ROS) production and reducing highly oxidized ROS. No significant difference (*p* ≤ 0.05) was observed in total phenolics content between the treated and the control watermelons (Fig. 7A). The radical scavenging activity of all the samples was measured using the 2,2-diphenyl-1-picrylhydrazyl (DPPH) assay. Nano-treatment had no detrimental effect on radical scavenging activity (Fig. 7B).

The studied priming treatments had no significant impact on mineral element contents of diploid and triploid watermelon fruit compared with their respective controls (Supplementary Information, Fig. S4). These results indicate that TNE and AgNP treatment did not cause any negative effect on nutritional quality of watermelon fruit.

## Discussion

### Nanopriming stimulated germination of watermelon seeds by modulating primary metabolites

The major form of carbohydrate is soluble sugar and the alteration of soluble sugars in germinating seeds can be a good indicator of seedling emergence and growth. Soluble sugars are mobilized from seeds to various organs in the seedlings during seed germination and early seedling growth^21^. Application of AgNPs enhanced the levels of glucose and fructose at 96 h after priming compared to those in unprimed diploid and triploid seeds (Fig. 2). Although sucrose levels decreased during the course of seed germination, the abundance of other carbohydrates (glucose and fructose) increased, which could be due to the conversion of polysaccharides to monosaccharides. This suggests a substantial mobilization from reserves into glycolysis. The data are in agreement with results obtained by gas chromatography analyses of sugars in rice^22^ and *Arabidopsis thaliana* seeds^23^. These data corresponded well with the early protrusion of radicles and enhanced growth rate in AgNP-primed seeds compared to other treatments. The germination results agree with previous studies of watermelon, zucchini, and corn crops^24^, which showed that, that compared with untreated watermelon, the germination rate was enhanced at all the AgNP concentrations (0.05 to 2.5 mg/mL)^24^. Similarly, enhanced germination was observed using AgNPs in rice^25,26^ and *Boswellia ovalifoliolata*^27^.

In the present study, AgNPs and TNE showed great promise for agricultural applications to enhance the germination rate and growth of diploid and triploid watermelon plants as compared to the control unprimed seeds. In TNE, ar-turmerone is the major compound found in the byproduct from curcumin manufacture and this compound has antibacterial^28^ and antifungal properties^29^. As a result of the antibacterial properties of thymol, significant enhancement of soybean seed germination and plant growth was recorded in plants treated with thymol nanoemulsion^30^. Therefore, nanoscale ar-turmerone could be used as an antimicrobial and plant growth-promoting agent, causing higher seed germination compared to the control treatment. Some dose-dependent negative results were reported using chemically synthesized AgNPs in wheat seedlings^31^, which demonstrate the toxicity of the reducing and capping agent. Therefore, green nanoparticles capped with phytochemicals could be benign alternatives for agricultural purposes.

### Nanopriming modulated the photosynthetic pigments and seedling growth

Chlorophyll is positively correlated with photosynthetic rate and the total photosynthate produced^32^. Enhanced chlorophyll content in AgNP-primed seedlings can be attributed to the increase in water and nutrient absorption, leading to better plant growth and physiological development. The increased content of chlorophyll observed in the AgNP-treated seedlings in this study could facilitate the higher accumulation of soluble proteins for plant metabolic activities, which can maintain higher physiological performance. Higher chlorophyll contents could accelerate the rate of photosynthetic CO_2_ fixation and subsequently produce more soluble sugars resulting higher biomass of the plants^32^. In treated seedlings of *Brassica juncea*, AgNPs improved the cellular electron exchange efficiency and photosynthetic quantum efficiency, and increase chlorophyll contents, as compared to the control seedlings^33^. The enhanced shoot length and stem diameter of watermelon transplants at 21 DAS could be attributed to the higher rate of photosynthesis in the treated plants. A similar result was obtained in the seedling growth of corn, watermelon, zucchini and pearl millet plants treated with AgNPs^24,34^.

Studies on the interactions between chlorophyll and NPs are imperative for understanding the photophysical behavior of plants exposed to NPs. To understand the potential toxicity of AgNPs and AgNO_3_ in seed priming, we measured chlorophyll in the leaf tissue of 14-day-old watermelon seedlings collected from the third-year emergence study. The chlorophyll content significantly decreased in AgNO_3_-treated seedlings, as compared to AgNPs, when the same concentration was used. AgNP priming had no significant impact on chl *a* and *b* contents when comparing unprimed and hydroprimed seed. Based on these results, we conclude that when used in the same concentrations, AgNO_3_ bulk treatment is phytotoxic but the synthesized AgNPs are not (Supplementary Information, Fig. S5). In comparative phytotoxicity studies, our group has demonstrated the negative impacts of bulk Fe particles as compared to the Fe nanoparticles on chlorophyll synthesis of watermelon seedlings^11^. Our results are in agreement with similar studies by other groups, which have demonstrated the inhibitory effect of bulk silver particles as compared to silver nanoparticles^26,35,36^. These studies confirmed the phytotoxic effects of silver are not due to nanoparticles but owing to the Ag^+^ ions. Indeed, Ag accumulation in seeds primed with AgNPs was lower than those of AgNO_3_ and this higher Ag accumulation inhibits seed germination and seedling growth in rice^26^, onion^37^, castor^35^, maize^38^, poplar, and Arabidopsis^39^. Differential toxicity of AgNPs and Ag^+^ (AgNO_3_) could be due to the slow surface generation of ROS by AgNPs in contrast to bulk particles^40^.

### Nanopriming enhanced growth and yield while maintaining nutritional quality

AgNP-treated watermelon vines had longer and thicker vines as compared to the control and TNE-treated watermelons. Significant differences in yield were also observed in AgNP-treated watermelon of both cultivars at Snook, as well as non-significant increases in Grapeland and Weslaco. The results are in agreement with studies on onion^41^, *Brassica juncea*^33^, common beans, corn^42^, safflower cultivars^43^, rice^26^, and wheat^44^. AgNPs increased plant growth and biochemical attributes of *Brassica juncea*^33^, and seed yield of safflower cultivars^43^. Seed priming with iron oxide nanoparticles in field crops such as wheat has been shown to be an effective method of increasing yields, and retaining quality, including the nutritive value of grains^44^. In the present study, nanoparticle-treated seeds germinated and grew very well as compared to the unprimed controls (Fig. 4). Moreover, AgNPs accumulated in the seeds (Fig. 6), which might activate the metabolic events that are vital for the seed germination and seedling growth. The pathway of nanoparticle transport through the xylem and phloem in corn, tomato, and watermelon has been verified previously^45–47^. Internalized nanoparticles are transported through the vascular system of the phloem and could induce gene expression^48^ resulting in the enhanced physiological parameters and ultimately productivity.

Along with the growth and the production of the crop, nutritional quality is a major concern. Interestingly, in our study, DPPH and total phenolics in nano-treated and untreated watermelons remained the same, which is in accordance with the literature on cerium oxide-treated medium amylose rice varieties^49^. Similarly, there were no significant differences in the level of macroelements (N, P, S, K, Ca, Na, Mg) and microelements (Fe, Cu, Mn, B, Zn) among the treatments. These findings suggest that the treatment of watermelon seeds with the nanoparticles would be unlikely to induce any negative effects on the nutritional quality of the crop.

## Conclusions

The present study presented a simple, low-cost, and ecofriendly approach to synthesize NPs using agro-industrial byproducts and avoids the use of hazardous and toxic chemicals for direct applications in agriculture. The TNE promoted germination and growth, but AgNPs hold greater promise for agricultural applications. Multiple lab, greenhouse, and field studies suggested that germination and growth parameters of watermelon seeds were enhanced after treatment with nanoparticles. Given the lowdosage of AgNPs used in this work, the material cost for commercial application is about $3-5/acre, making this a cost-efficient seed treatment method. Therefore, nanopriming could be cost-effective for seed priming and can support sustainable development of agriculture as well as improve the socio-economic status of farmers. Nanoparticle-mediated seed treatments avoid the release of large amounts of nanomaterials into the field, lowering impacts on the environment. Moreover, discarding industrial by-products aggravates disposal problems in the environment; however, these by-products are used in the present study for the synthesis of nanoparticles. To further enhance the effectiveness of the nanomaterial treatments, future efforts should focus on optimization of priming time, the concentration of the priming solution and nanomaterial composition, structure, and activity. Similarly, plant physiology affects the interaction with nanoparticles, so results observed in one crop are not necessarily valid for other crops, which makes it imperative to study different plant species. Therefore, the results of these studies should stimulate investigations to understand nanoparticle–plant surface interactions and the uptake of the green nanoparticles in the plant system.

## Supplementary information

Supplementary information
