# Supplementary material for: Nanoparticle-Mediated Seed Priming Improves Germination, Growth, Yield, and Quality of Watermelons (Citrullus lanatus) at multi-locations in Texas
Source: Sci Rep. 2020 Mar 19;10:5037. doi: 10.1038/s41598-020-61696-7 (PMC7081193; doi:10.1038/s41598-020-61696-7)
Supplement: Supplementary file 1 — Supplementary information [file 41598_2020_61696_MOESM1_ESM.docx]

**Nanoparticle-Mediated Seed Priming Improves Germination, Growth, Yield, and Quality of Watermelons (*Citrullus lanatus*) at multi-locations in Texas**

Pratibha Acharya^1^, G. K. Jayaprakasha^1,^*, Kevin M. Crosby^1^, John L. Jifon^1,2^ and

Bhimanagouda S. Patil^1,^*

*^1^Vegetable and Fruit Improvement Center, Department of Horticultural Sciences, Texas A&M University, 1500 Research Parkway, A120, College Station, TX 77845-2119, United States*

*^2^Department of Horticultural Sciences, Texas A&M AgriLife Research and Extension Center, 2415 E Hwy 83, Weslaco, TX, 78596, United States*

*Correspondence

Phone: +1 979-458-8090; Fax: +1 979-862-4522; E mail: [b-patil@tamu.edu](mailto:b-patil@tamu.edu); [gkjp@tamu.edu](mailto:gkjp@tamu.edu)

**Figure S1**. Map of Texas, USA with all the growing environment conditions. First year, watermelon plants were grown at four locations of Texas; Edinburg (26°18′15″N 98°9′50″W), Snook (30°29′25″N 96°28′11″W), Pecos (31°24′56″N 103°30′0″W), and Grapeland (31°29′30″N 95°28′49″W) in the grower’s field during the summer season of 2016/17. Moreover, in order to validate the result of the first year, another field trial was conducted in Texas A&M AgriLife Research and Extension Center at Weslaco (26º15’N, 97º98’W) in 2017/18.

TAMU: Texas A&M University, College Station where all the harvested samples were collected and analyzed.

**Figure S2**. Rainfall (inches), maximum and minimum temperature (°C) of all the growing environments. During harvesting time (June) in Weslaco, there was heavy storm and rainfall that led to flooding in the watermelon fields so we were able to harvest only once.

Source: http://www.texmesonet.org/HistoricalData

**Figure S3.** Histograms containing mean particle size (PS), polydispersity index (PDI) and the zeta potential (ZP) from photon correlation spectroscopy for (A) turmeric nanoemulsion (B) silver nanoparticles, (C) UV-Vis spectra of silver nanoparticles. A single, strong, and broad surface plasmon resonance (SPR) peak was observed for silver nanoparticles at 410 nm. Data represent mean ± SEM (n = 3).


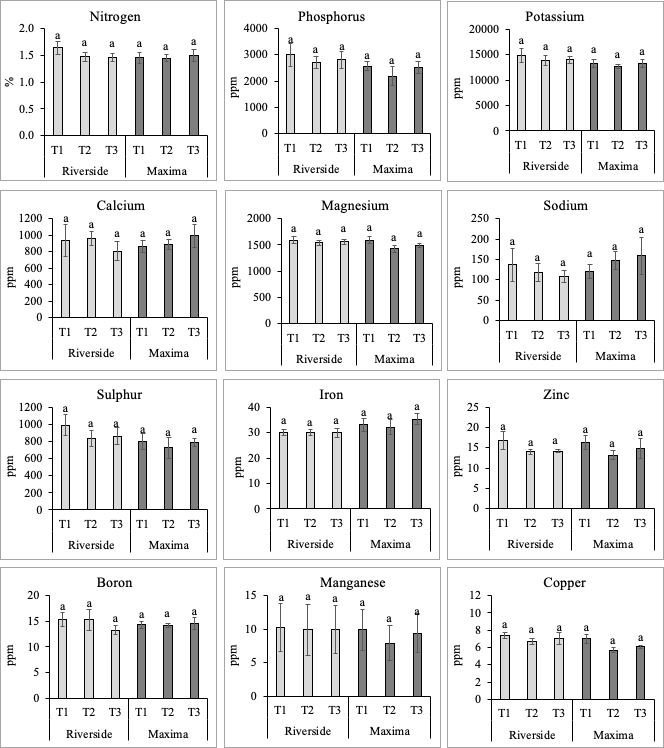


**Figure S4.** Elemental analysis of nanoparticle treated and untreated watermelon fruits. T1: unprimed, T2: turmeric oil nanoemulsion and T3: silver nanoparticle. Same letters above a bar indicate there was no significant difference at (*p* ≤ 0.05) between the treatments. Data represent mean ± SEM (n = 15).

**Figure S5.**  Influence of seed priming treatments on the levels of chlorophyll *a* and *b* of 14-day-old Riverside watermelon seedlings. Values are average ± standard error of three replicates. The post hoc test significant differences (*p* ≤ 0.05) among different treatments are shown by different letters. UP, unprimed; HP, hydroprimed; TNE, turmeric oil nanoemulsion; AgNO_3_; silver nitrate; and AgNPs, silver nanoparticles.

| S. No | 2θ (degrees) | FWHM (β) (radians) | d-spacing (°A) | Miller indices | Crystallite size (nm) |
| --- | --- | --- | --- | --- | --- |
| 1 | 38.19 | 0.118 | 2.354 | 111 | 47.5 |
| 2 | 44.38 | 0.156 | 2.04 | 200 | 39.7 |
| 3 | 64.49 | 0.186 | 1.443 | 220 | 33.3 |
| 4 | 77.45 | 0.277 | 1.231 | 311 | 25.3 |
|  |  |  |  | Average size (D) = 36.5 | |

**Table S1.** Calculation of the average crystallite size of AgNPs synthesized with onion peel extracts, using Debye Scherer’s equation.

| Analysis | Units | 2017 | | | | 2018 |  |
| --- | --- | --- | --- | --- | --- | --- | --- |
|  |  | Grapeland | Edinburg | Pecos | Snook | Weslaco | Weslaco |
| pH | - | 5.9 | 8.5 | 8 | 7.9 | 7.9 |  |
| Conductivity | umho/cm | 75 | 129 | 2240 | 892 | 367 |  |
| Nitrate-N | ppm | 8 | 4 | 207 | 124 | 23 |  |
| Phosphorus | ppm | 39 | 39 | 61 | 121 | 54 |  |
| Potassium | ppm | 30 | 203 | 1112 | 419 | 387 |  |
| Calcium | ppm | 314 | 4484 | 9974 | 4783 | 3594 |  |
| Magnesium | ppm | 34 | 150 | 676 | 156 | 307 |  |
| Sulfur | ppm | 3 | 879 | 23 | 3 | 51 |  |
| Sodium | ppm | 8 | 264 | 34 | 1 | 99 |  |

**Table S2.** Soil analysis of growing environment conditions for both years. Soil samples were analyzed by the Soil, Water and Forage Testing Laboratory, Department of Soil and Crop Sciences, Texas A&M University, College Station, TX.

| Cultivar | Nanopriming solution | Compound | Control seed | Treated seed (μg/g FW) |
| --- | --- | --- | --- | --- |
| Riverside | Turmeric oil nanoemulsion | Ar-Turmerone | N.D. | 2250.18 ± 67.65 |
|  | Silver nanoparticles | Silver | N.D.* | 20.86 ± 7.21 |
| Maxima | Turmeric oil nanoemulsion | Ar-Turmerone | N.D. | 2422.46 ± 111.95 |
|  | Silver nanoparticles | Silver | N.D.* | 15.63 ± 3.84 |

**Table S3.** Internalization of silver nanoparticles (μg/g FW) and turmeric nanoemulsion in treated Riverside (diploid) and Maxima (triploid) watermelon seeds. * value was below detection limit of INAA analysis (40 ng/g for Ag).

Control and silver nanoparticle (AgNPs) primed watermelon seeds were tested by instrumental neutron activation analysis to determine the silver concentration. Turmeric oil nanoemulsion treated watermelon seeds were tested by GC-MS analysis to determine the active compound. Ar- Turmerone is the major compound found in turmeric oil nanoemulsion. Values are means of three replicates ± SEM.
